# Genetic background of walking ability and its relationship with leg defects, mortality, and performance traits in turkeys (*Meleagris gallopavo*)

**DOI:** 10.1016/j.psj.2024.103779

**Published:** 2024-05-09

**Authors:** Henrique A. Mulim, Rick O. Hernandez, Ryley Vanderhout, Xuechun Bai, Owen Willems, Prafulla Regmi, Marisa A. Erasmus, Luiz F. Brito

**Affiliations:** ⁎ Department of Animal Sciences, Purdue University, West Lafayette, IN, USA; † Hendrix Genetics Limited, Kitchener, ON, Canada; ‡ Department of Poultry Science, University of Georgia, Athens, GA, USA

**Keywords:** animal welfare, leg deformity, poultry, productive trait

## Abstract

This study aimed to explore the genetic basis of walking ability and potentially related performance traits in turkey purebred populations. Phenotypic, pedigree, and genomic datasets from 2 turkey lines hatched between 2010 and 2023 were included in the study. Walking ability data, defined based on a scoring system ranging from 1 (worst) to 6 (best), were collected on 192,019 animals of a female line and 235,461 animals of a male line. Genomic information was obtained for 46,427 turkeys (22,302 from a female line and 24,125 from a male line) using a 65K single nucleotide polymorphism (**SNP**) panel. Variance components and heritability for walking ability were estimated. Furthermore, genetic and phenotypic correlations among walking ability, mortality and disorders, and performance traits were calculated. A genome-wide association study (**GWAS**) was also conducted to identify SNPs associated with walking ability. Walking ability is moderately heritable (0.23 ± 0.01) in both turkey lines. The genetic correlations between walking ability and the other evaluated traits ranged from -0.02 to -0.78, with leg defects exhibiting the strongest negative correlation with walking ability. In the female line, 31 SNPs were associated with walking ability and overlapped with 116 genes. These positional genes are linked to 6 gene ontology (**GO**) terms. Notably, genes such as *CSRP2, DDX1, RHBDL1, SEZ6L*, and *CTSK* are involved in growth, development, locomotion, and bone disorders. GO terms, including fibronectin binding (GO:0001968), peptide cross-linking (GO:0018149), and catabolic process (GO:0009057), are directly linked with mobility. In the male line, 66 markers associated with walking ability were identified and overlapped with 281 genes. These genes are linked to 12 GO terms. Genes such as *RB1CC1, TNNI1, MSTN, FN1, SIK3, PADI2, ERBB4, B3GNT2*, and *BACE1* are associated with cell growth, myostatin development, and disorders. GO terms in the male line are predominantly related to lipid metabolism. In conclusion, walking ability is moderately heritable in both populations. Furthermore, walking ability can be enhanced through targeted genetic selection, emphasizing its relevance to both animal welfare and productivity.

## INTRODUCTION

Genetic selection has significantly contributed to improve turkey (*Meleagris gallopavo*) productivity, resulting in heavier animals with increased body size and improved meat yield (Soyalp et al., 2023). Although this progress is beneficial for production, it has led to disorders associated with rapid growth, such as aortic rupture, tibial dyschondroplasia, footpad dermatitis, and other leg disorders (Hafez and Shehata, 2021), which affect walking ability, increasing mortality, and negatively impacting animal welfare and the economic viability of the turkey industry. Leg health and mobility issues are major challenges in commercial turkey production, representing concerns for both animal welfare and profitability (Dong et al., 2023). This economic impact coincides with increased consumer concern about the welfare and health of poultry, necessitating that modern and sustainable poultry production comply with acceptable welfare standards for animals (Marchewka et al., 2020). Therefore, understanding the genetic and phenotypic relationships between body weight, mobility and leg health, and the identification of novel selection traits become imperative to ensure that animals can effectively support the production demands while experiencing acceptable levels of animal welfare.

Genetic selection for walking ability has been proposed as an indicator trait, offering insights into bird mobility and enabling correction for certain leg-related abnormalities (Soyalp et al., 2023). Walking ability is a compound index based on different traits such as walking motion, pitch, balance, leg angulation, hock and hip strength, and leg structure that can be evaluated by a human observer who classifies individuals into levels ranging from poor (score 1) to good (score 6) (Quinton et al., 2011). For instance, the scoring system described by Quinton et al. (2011) is used in practice in the turkey industry to select turkeys for breeding programs, and in this scoring system, a turkey with a score of 6 must exhibit fluid mobility, excellent pitch, balance, low outward leg angulation, no hock and hip weakness, and no leg defects. Nevertheless, assessing the relationship between walking ability and fast growth-related problems in turkeys requires a deeper understanding of their genetic background to validate its use as an indicator trait. Therefore, the primary objectives of this study were to: 1) estimate variance components and genetic parameters for walking ability in 2 pure lines of turkeys (one female and one male line); 2) assess phenotypic and genetic correlations between walking ability and performance traits, mortality and disorder traits; 3) conduct a genome-wide association study to identify genomic markers significantly associated with walking ability in both populations; and, 4) perform gene annotation and functional enrichment analyses of the genomic regions and genes found to be associated with walking ability. The overarching aim of this study is to contribute to a better understanding of the genetic basis of walking ability in turkeys, which can inform future breeding strategies for improving overall turkey welfare and production efficiency.

## MATERIAL AND METHODS

Approval from the Animal Care Committee was not needed for this study since all analyses were conducted using pre-existing databases. This study utilized 2 distinct lines of purebred Turkeys (*Meleagris gallopavo*), 1 male line and 1 female line. Phenotypic records, pedigree, and genomic information were provided by Hendrix Genetics (Kitchener, ON, Canada). Phenotypic records deviating 3.5 standard deviations or more from the mean were excluded from further analyses. A summary of the traits evaluated is presented in Table 1.

**Table 1:** Descriptive statistics of performance traits and walking ability (**WALK**) for female and male lines of turkeys: number of observations (**n**), minimum (**min**), maximum (**max**), mean, and standard deviation (**SD**) for each trait.

			n	min	max	mean	SD
Female line	*Performance trait*	Body weight 1					
		*Female*	8,357	5,650.00	8,600.00	7,105.12	540.19
		*Male*	158,042	5,600.00	10,800.00	8,212.32	945.42
		Body weight 2					
		*Female*	137,321	7,750.00	11,350.00	9,540.30	638.77
		*Male*	103,663	12,350.00	20,600.00	16,474.70	1,468.42
		Breast meat yield					
		*Male*	23,879	17.47	36.42	24.44	1.82
		Live weight					
		*Male*	30,238	14,450.00	2,260.00	18,563.56	1,480.32
		Egg production					
		*Female*	19,178	12.00	164.00	90.13	25.82
	WALK	*Female*	90,389	1	6	2.25	0.84
		*Male*	101,630	1	6	1.17	0.71
Male line	*Performance trait*	Body weight 1					
		*Female*	180,054	7,100	11,900	9,511.18	860.93
		*Male*	176,664	7,150	14,750	10,963.07	1,364.28
		Body weight 2					
		*Female*	144740	10250	15,850	13,053.57	1,019.27
		*Male*	113801	18480	29,050	23,751.31	1,901.59
		Breast meat yield					
		*Male*	23361	18.067	30.854	24.46	2.31
		Live weight					
		*Male*	30,056	18,800	29,600	24,288.69	1,964.70
		Egg production					
		*Female*	18,538	0	126	53.089	23.69
	WALK	*Female*	123,921	1	6	2.22	0.83
		*Male*	111,540	1	6	1.76	0.71

The study included animals hatched between 2010 and 2023. Three weight measures were considered: body weight 1 (in grams) recorded from 11 to 15 wk of age for males and 12 to 15 wk for females; body weight 2 (in grams) measured from 19 to 23 wk of age for males and 17 to 21 wk of age for females; live weight (in grams) recorded only for males, correspond to the weight of the animals at slaughter, measured between 16 and 24 wk of age; percentage of breast meat yield (in grams) was determined by dividing the breast weight by the live weight at slaughter, multiplied by 100; total egg production was assessed for females between 29 and 64 wk of age; and, walking ability was evaluated by trained technicians on a scale of 1 to 6 using the protocol described by Quinton et al. (2011), where a score of 1 indicated poor walking ability and a score of 6 indicated good walking ability.

For the mortality and disorder traits, the dataset with binary records for the female line (recorded on male and female animals) consisted of 193,508 observations. The breakdown of the number of animals affected by each trait is as follows: 29,622 (15.31%) for mortality (11,200 females and 18,422 males), 27,274 (14.09%) for other defects (14,907 females and 12,367 males), 10,557 (5.45%) euthanized (4,688 females and 5,869 males), and 126,055 (65.14%) with leg defects (46,075 females and 79,980 males). For the male line (also recorded on male and female animals), the dataset consisted of 233,354 phenotypic observations. The number of animals presenting by each trait is as follows: 50,657 (21.71%) for mortality (17,618 females and 33,039 males), 33,749 (14.46%) for other defects (12,991 females and 20,758 males), 14,893 (6.38%) euthanized (5,895 females and 8,998 males), and 134,055 (57.45%) with leg defects (50,904 females and 83,151 males).

### Genotypic Information

A total of 50,486 animals (25,531 from the female line and 24,955 from the male line) were genotyped utilizing a proprietary 65K single nucleotide polymorphism (**SNP**) chip panel (Illumina, Inc., San Diego, CA). The genotyping quality control criteria included filtering out SNPs with minor allele frequency (**MAF**) lower than 0.01, sample and SNP call rate lower than 90%, and extreme departure from Hardy-Weinberg equilibrium (**HWE**) higher than 0.15 (as an indication of genotyping errors). Additionally, markers with unknown genomic positions or those located in nonautosomal regions were excluded from further analyses. Quality control was performed within line and 22,302 animals and 51,138 SNPs remained for the female line while 24,125 animals and 51,352 SNPs were kept for the male line.

### Variance Components and Genetic Parameters

Variance component analyses were performed separately for each line as they are lowly genetically related (i.e., low genetic connectedness) and have been genetically selected for different traits (or similar traits but different weights in the selection indexes) over time. Variance components for walking ability and performance traits were estimated based on linear mixed animal models and the Average Information Restricted Maximum Likelihood (**AIREML**) algorithm. On the other hand, mortality, leg defects, euthanized, and other defects were analyzed using a mixed threshold-linear model due to their binary (1 – nonaffected or 2 - affected) recording. The analyses were done using the BLUPF90+ family of programs, including BLUPF90+, GIBBSF90+, and POSTGIBBSF90 (Misztal et al., 2014; Lourenco et al., 2020). For threshold analyses, 500,000 cycles were executed with a burn-in period of 100,000 cycles and thinning of 100 samples. The assessment of convergence and posterior inference was conducted using the “boa” R package (Smith, 2007).

The mixed model equations were solved based on the single-step GBLUP approach [**ssGBLUP**; Misztal et al., (2009)]. This method integrates the pedigree with genomic relationship matrices in a hybrid relationship matrix calculated as (Aguilar et al., 2010):

$$H^{-1}=A^{-1}+\left[\begin{array}{cc} 0 & 0\\ 0 & G^{-1}-\;A_{\;22}^{-1} \end{array}\right]$$

where **A** is the pedigree-based relationship matrix for all individuals in each turkey line, **A**_22_ is the pedigree-based relationship matrix of the genotyped animals for each line, and **G** is the genomic relationship matrix calculated as (VanRaden, 2008):

$$G={\mathrm{ZZ}}^{\prime }$$

where **Z** represents a matrix containing adjustments for allelic frequencies. These adjustment factors were incorporated to align the mean diagonal of matrix **G** with **A_22_**, ensuring a close correspondence between the 2 matrices (Vitezica et al., 2011).

The following single-trait animal model fitted can be described as:

y=Xb+Za+e

where **y** is the vector of phenotypic values; **b** is the vector of systematic effects of hatch (week and year that the animal was born), sex (with exception of the total egg production, breast meat yield, and live weight traits, which were recorded in a single sex), and age at measurement in weeks as a linear covariate for total egg production, mortality and disorder traits; **a** is the vector of random additive genetic effects; **e** is the vector of random residual errors; **X**, and **Z** are the incidence matrices associated with the systematic and additive genetic effects, respectively. The residuals were assumed to be independent and follow a normal distribution with a mean of zero and a variance of **I**σ²_e_. Additive genetic effects were also assumed to follow a normal distribution with a mean of 0 and a variance of **H**σ²_a_. From the variance components obtained, the narrow-sense heritability (h^2^) estimates were calculated as:

$${\hat{h}}^{2}=\frac{{\hat{\sigma }}_{a}^{2}}{{\hat{\sigma }}_{a}^{2}+{\hat{\sigma }}_{e}^{2}}$$

For the categorical traits (mortality and disorder traits) the heritability estimates were presented on the liability scale.

### Phenotypic and Genetic Correlations

The correlations among traits were calculated based on bivariate analyses using the AIREML method for performance traits and walking ability, and Bayesian methods when the traits were estimated together with the mortality and disorder traits. The analyses were done using the BLUPF90+ family programs (BLUPF90+, GIBBSF90+, and POSTGIBBSF90 (Misztal et al., 2014; Lourenco et al., 2020). For the threshold analyses, 500,000 cycles were performed with a burn-in phase of 100,000 cycles and a thinning process of 100. Convergence assessment and posterior inference were checked through the implementation of the “boa” R package (Smith, 2007). The following bivariate animal model was applied:

$$\left[\begin{array}{c} y_{1}\\ y_{2} \end{array}\right]=\left[\begin{array}{cc} X_{1} & 0\\ 0 & X_{2} \end{array}\right]\left[\begin{array}{c} b_{1}\\ b_{2} \end{array}\right]+\left[\begin{array}{cc} Z_{1} & 0\\ 0 & Z_{2} \end{array}\right]\left[\begin{array}{c} a_{1}\\ a_{2} \end{array}\right]+\left[\begin{array}{c} e_{1}\\ e_{2} \end{array}\right]$$

Where **y_1_** and **y_2_** are the vectors of phenotypic records for traits 1 and 2, respectively; **b_1_** and **b_2_** are the vectors of systematic effects as defined for the single trait analyses; **a_1_** and **a_2_** are the vectors of direct additive genetic effects for traits 1 and 2, respectively; **e_1_** and **e_2_** are the vectors of residual errors for traits 1 and 2, respectively; **X** and **Z** are the incidence matrices associated with the systematic and additive genetic effects, respectively. The residuals were assumed to be independent and normally distributed with a mean of zero and a variance of **I**σ²_e_. Additive genetic effects were also assumed to follow a normal distribution with a mean of 0 and a variance of **H**σ²_a_. The magnitude of the phenotypic and genotypic correlations are discussed as suggested by Bourdon (1997).

### Genome-Wide Association Study for Walking Ability

The single-step genome-wide association study (**ssGWAS**) was employed using the BLUPF90+ family of programs (Misztal et al., 2014; Lourenco et al., 2020) and the approach described by Wang et al. (2012). Approximate *P*-values were calculated as proposed by Aguilar et al. (2019). We applied the Bonferroni correction method (alpha = 0.05) to adjust for multiple testing based on the number of independent chromosomal segments as proposed by Corbin et al. (2012), the average length of a chromosome and the number of chromosomes at the chromosome wide level. This correction method accounts for multiple testing and adjusts the significance thresholds of the SNPs accordingly (Goddard et al., 2011).

### Gene Annotation and Functional Enrichment Analyses

For the annotation of the SNP associated with walking ability, we used the GALLO package (Fonseca et al., 2020) and considered a genomic window spanning 100 Kb upstream and downstream of the significant SNPs. The function and association of each gene was verified on the GeneCard platform (Safran et al., 2010). Subsequently, the positional genes identified through the annotation process were subjected to functional analyses using the *gprofiler2* package (Kolberg et al., 2020). These analyses enabled the identification of biological processes (**BP**), molecular functions (**MF**), and cellular components (**CC**) in which the identified candidate genes are involved.

## RESULTS

### Variance Components, Heritability, and Genetic Correlations

The variance components and heritability estimates for walking ability, performance traits, and mortality and disorder traits measured in the female line are presented in Table 2.

**Table 2:** Variance components and heritability estimates for productive traits, mortality and disorder traits, and walking ability in a turkey female line.

Trait	σ^2^_a_	PSD/SE	σ^2^_e_	PSD/SE	σ^2^_p_	PSD/SE	h^2^	PSD/SE
Body weight 1	224,720.00	4,859.70	167,570.00	2,389.00	392,290.00	2,855.60	0.57	0.01
Body weight 2	423,940.00	9,395.20	522,830.00	4,633.10	946,770.00	5,691.70	0.45	0.01
Breast meat yield	0.73	0.05	1.70	0.03	2.43	0.03	0.30	0.02
Live weight	501,650.00	24,095.00	737,050.00	15,119.00	1,238,700.00	14,239.00	0.40	0.02
Egg production	45.40	2.82	122.41	1.78	167.80	2.28	0.27	0.01
Mortality	0.10	0.01	1.00	0.00	1.10	0.01	0.09	0.01
Other defects	0.09	0.01	1.00	0.00	1.09	0.01	0.08	0.01
Euthanized	0.31	0.02	1.00	0.00	1.31	0.02	0.24	0.01
Leg defects	0.14	0.01	1.00	0.00	1.14	0.01	0.12	0.01
Walking ability	0.14	0.00	0.48	0.00	0.62	0.00	0.23	0.01

σ^2^_a_: additive genetic variance; SE: standard errors for performance traits and walking ability (linear models); PSD: posterior standard deviations for mortality and disorder traits (Bayesian threshold models); σ^2^_e_: residual variance; σ^2^_p_: phenotypic variance; h^2^: heritability estimate.

The heritability estimates of the analyzed traits ranged from low (defects – 0.08 ± 0.01) to high (body weight 1 – 0.57 ± 0.01), with walking ability exhibiting a moderate heritability of 0.23 ± 0.01. Table 3 presents the genetic and phenotypic correlations among the traits for the female line.

**Table 3:** Heritability (diagonal), genetic correlation (above diagonal), and phenotypic correlation (below diagonal) among productive traits, mortality and disorder traits, and walking ability in a turkey female line.

	Body weight 1	Body weight 2	Breast meat yield	Live weight	Egg production	Mortality	Other defects	Euthanized	Leg Defects	WALK
Body weight 1	**0.57**	0.81	0.07	0.73	−0.06	−0.05	−0.08	−0.02	0.26	−0.36
Body weight 2	0.67	**0.45**	0.05	0.92	−0.41	0.00	−0.15	0.14	0.32	−0.46
Breast meat yield	0.10	0.13	**0.30**	0.01	0.10	−0.19	−0.12	−0.08	0.23	−0.42
Live weight	0.57	0.77	0.03	**0.40**	−0.49	0.01	−0.05	−0.13	0.09	−0.05
Egg production	−0.06	−0.21	-	-	**0.27**	0.76	−0.01	−0.08	0.28	0.12
Mortality	0.01	0.00	0.00	0.01	0.25	**0.09**	0.42	0.15	−0.73	0.44
Other defects	−0.13	−0.07	−0.01	0.01	0.00	−0.89	**0.08**	−0.20	−0.57	0.51
Euthanized	−0.02	−0.16	−0.01	0.49	−0.01	−0.80	−0.75	**0.24**	−0.46	0.33
Leg defects	0.06	0.08	0.01	−0.02	0.04	−0.84	−0.67	−0.69	**0.12**	−0.54
Walking ability	−0.18	−0.16	−0.13	−0.08	0.03	0.19	0.38	0.27	−0.74	**0.23**

Positive and negative as well as favorable and unfavorable genetic correlations, ranging from weak to strong associations, were observed among all the trait pairs. Walking ability is genetically and phenotypically correlated with most of the performance traits evaluated and ranged from -0.46 (walking ability and body weight 2) to 0.12 (walking ability and egg production). The only positive genetic correlation observed between a performance trait and walking ability was with egg production, in which a genetic and phenotypic correlation of 0.12 and 0.03, respectively, was observed between both traits. Mortality and disorder traits were moderately to strongly correlated with walking ability. More specifically, leg defects had a genetic correlation of -0.54 with walking ability, indicating that selection for improved walking ability is expected to reduce the incidence of leg defects. The standard errors and posterior standard deviations for the traits are presented in Supplementary Material 1.

Table 4 shows the variance components and heritability estimates for all the different traits measured in the male line.

**Table 4:** Variance components and heritability estimates for productive traits, mortality and disorder traits, and walking ability in a turkey male line.

		σ^2^_a_	PSD/SE	σ^2^_e_	PSD/SE	σ^2^_p_	PSD/SE	h^2^	PSD/SE
*Performance traits*	Body weight 1	412,440.00	7,696.60	559,800.00	3,948.50	972,240.00	4,584.40	0.42	0.01
	Body weight 2	637,340.00	13,371.00	1,033,900.00	7,186.60	1,671,200.00	8,201.10	0.38	0.01
	Breast meat yield	0.73	0.05	2.36	0.04	3.10	0.03	0.24	0.01
	Live weight	681,250.00	36,314.00	1,362,300.00	25,061.00	2,043,500.00	21,602.00	0.33	0.02
	Egg production	106.48	6.24	207.66	4.17	314.14	4.20	0.34	0.02
*Mortality and disorder traits*	Mortality	0.42	0.02	1.00	0.00	1.42	0.02	0.30	0.01
	Other defects	0.26	0.01	1.00	0.00	1.26	0.01	0.20	0.01
	Euthanized	0.51	0.03	1.00	0.00	1.51	0.03	0.34	0.01
	Leg Defects	0.13	0.01	1.00	0.00	1.13	0.00	0.12	0.01
	Walking ability	0.15	0.00	0.48	0.00	0.63	0.00	0.23	0.01

σ^2^_a_: additive genetic variance; SE: standard errors posterior standard deviation for productive traits and walkability; PSD: posterior standard deviation for mortality and disorder traits; σ^2^_e_: residual variance; σ^2^_p_: phenotypic variance; h^2^: heritability estimate.

The heritability estimates ranged from low (leg defects -0.12 ± 0.01) to high (body weight 1 – 0.42 ± 0.01), with walking ability with a heritability estimate of 0.23 ± 0.01. Table 5 presents the phenotypic and genetic correlations among performance traits, mortality, and disorder traits, and walking ability for the male line.

**Table 5:** Heritability (diagonal), genetic correlation (above diagonal), and phenotypic correlation (below diagonal) among productive traits, mortality and disorder traits, and walking ability in a turkey male line.

		*Performance traits*					*Mortality and disorder traits*				
		Body weight 1	Body weight 2	Breast meat yield	Live weight	Egg production	Mortality	Other defects	Euthanized	Leg defects	Walking ability
*Performance traits*	Body weight 1	**0.42**	0.91	0.00	0.77	−0.06	−0.03	−0.16	−0.02	0.32	−0.43
	Body weight 2	0.56	**0.38**	−0.04	0.70	−0.09	−0.06	−0.18	−0.03	0.25	−0.46
	Breast meat yield	0.15	0.11	**0.24**	0.02	0.20	−0.18	−0.20	−0.27	0.19	−0.18
	Live weight	0.67	0.33	0.11	**0.33**	0.01	−0.06	−0.03	−0.12	0.23	−0.30
	Egg production	−0.15	−0.16	-	-	**0.34**	0.05	−0.03	−0.08	0.32	−0.02
*Mortality and disorder traits*	Mortality	−0.03	−0.08	0.68	0.25	−0.22	**0.30**	−0.61	−0.43	−0.77	0.47
	Other defects	−0.16	−0.13	−0.02	−0.04	−0.01	−0.71	**0.20**	0.49	−0.40	0.50
	Euthanized	−0.04	−0.03	0.67	0.11	−0.01	−0.86	−0.41	**0.34**	−0.58	0.42
	Leg defects	0.07	0.05	−0.15	−0.03	0.03	−0.92	−0.62	−0.56	**0.12**	−0.76
	Walking ability	−0.15	−0.16	0.12	−0.10	0.00	0.67	0.61	0.52	−0.78	**0.23**

The genetic correlations among the traits were variable. Walking ability is negatively and unfavorably genetically correlated with all performance traits and ranged from -0.46 (walking ability and body weight 2) to -0.02 (walking ability and egg production). At the phenotypic level, most traits were weakly and negatively correlated with walking ability, except egg production which showed a null correlation with walking ability. The standard errors and posterior standard deviations for the traits are presented in Supplementary Material 2.

### Genome-Wide Association, Gene Annotation, and Functional Analyses

**Figure 1:**
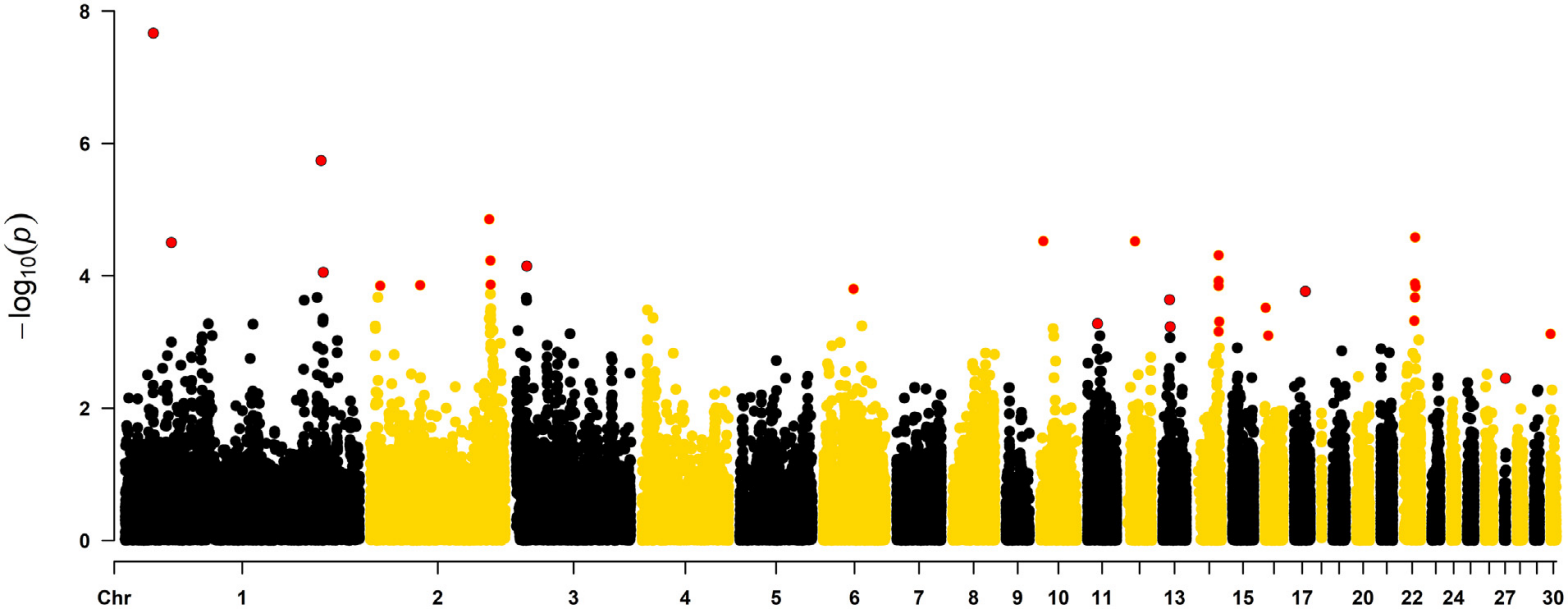
Genome-wide association study for walking ability in a female turkey line population. Red dots indicate the significant markers at the chromosome-wise level.

Figure 1 shows the Manhattan plot for the GWAS for walking ability in the female line.

In total, 31 markers located on 14 chromosomes (1, 2, 3, 6, 10, 11, 12, 13, 14, 16, 17, 22, 27, and 30) were statistically associated with walking ability. The genome location, significance, and frequency of the markers are presented in Table 6.

**Table 6:** Significant markers associated with walking ability in a female line of turkeys.

Chromosome	Position	*P*-value	Allele frequency
1	22,890,949	2.14^−08^	0.66
1	37,542,076	3.14^−05^	0.12
1	159,380,501	1.81^−06^	0.64
1	161,195,255	8.85^−05^	0.85
2	7,930,470	1.41^−04^	0.06
2	40,372,989	1.39^−04^	0.54
2	96,638,915	1.41^−05^	0.60
2	97,549,801	5.88^−05^	0.27
2	97,757,157	1.36^−04^	0.64
3	8,515,542	7.16^−05^	0.21
6	24,567,125	1.58^−04^	0.20
10	2,298,097	2.99^−05^	0.10
11	8,129,556	5.26^−04^	0.08
12	5,949,817	3.01^−05^	0.45
13	5,522,922	2.31^−04^	0.70
13	6,073,166	5.88^−04^	0.70
14	17,271,637	6.92^−04^	0.36
14	17,277,317	4.90^−05^	0.34
14	17,286,509	1.20^−04^	0.72
14	17,290,496	1.43^−04^	0.33
14	17,837,604	4.93^−04^	0.05
16	581,156	3.06^−04^	0.90
16	2,758,958	7.99^−04^	0.87
17	8,961,744	1.71^−04^	0.06
22	8,568,573	4.79^−04^	0.08
22	8,971,663	2.13^−04^	0.16
22	9,056,038	1.31^−04^	0.12
22	9,139,612	2.62^−05^	0.11
22	9,473,232	1.47^−04^	0.14
27	1,149,349	3.53^−03^	0.57
30	29,693	7.54^−04^	0.63

The significant markers overlapped with 116 genes, which have been classified as protein-coding (104), long noncoding RNAs (9), and pseudogenes (3). The genes annotated for walking ability in the female line are presented in Supplementary Material 3.

From the genes annotated, some of them were related to growth and cell development (e.g., *CSRP2* [Cysteine and glycine-rich protein 2], *DDX1* (DEAD-Box Helicase 1)*,* and *RHBDL1* [Rhomboid Like 1]), locomotion (e.g., *SEZ6L -* Seizure Related 6 Homolog Like)*,* and bone and muscular disorder (e.g., *CTSK* [Cathepsin K], *B3GNT2* [beta-1,3-N-acetylglucosaminyltransferase]). The gene ontology (**GO**) terms in which these genes are involved are presented in Table 7. In summary, 2 biological processes and 4 molecular functions are associated with the genes found to be related to walking ability in the female line.

**Table 7:** Gene ontology terms for the genes annotated for walking ability in turkey's female line.

Term ID	Source	Term name	*P*-value
GO:0018149	GO:BP	Peptide cross-linking	2.18^−03^
GO:0009057	GO:BP	Macromolecule catabolic process	2.36^−02^
GO:0003810	GO:MF	Protein-glutamine gamma-glutamyltransferase activity	3.80^−05^
GO:0004175	GO:MF	Endopeptidase activity	4.34^−02^
GO:0001968	GO:MF	Fibronectin binding	4.70^−02^
GO:0008233	GO:MF	Peptidase activity	4.70^−02^

Abbreviations: BP, biological process; MF, molecular function.

From the GO terms significantly related to the genes associated with walking ability, 2 important GO terms related to the femoroacetabular joint were identified: the peptide cross-linking (GO:0018149) and the fibronectin binding (GO:0001968).

**Figure 2:**
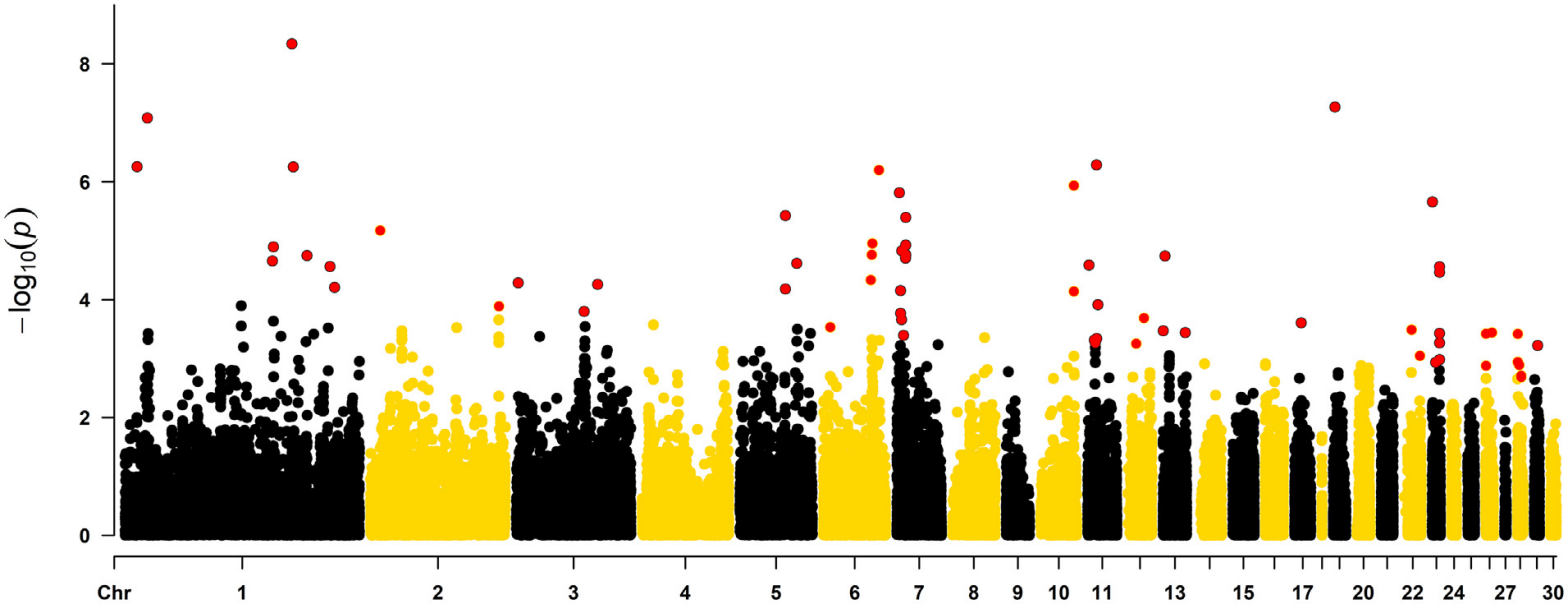
Genome-wide association study for walking ability in a male turkey line population. Red dots indicate the significant markers at the chromosome-wise level.

Figure 2 shows the Manhattan plot of the GWAS for walking ability in the male line population.

Sixty-six markers located on 17 chromosomes (1, 2, 3, 5, 6, 7, 10, 11, 12, 13, 17, 19, 22, 23, 26, 28, and 29) were shown to be associated with walking ability on the male line. The genomic position and allele frequency are presented in Table 8.

**Table 8:** Significant markers associated with walking ability in the male line of turkeys.

Chromosome	Position	*p*-value	Allele Frequency
1	9,581,988	5.53^−07^	0.46
1	18,042,125	8.22^−08^	0.76
1	119,774,977	2.20^−05^	0.07
1	120,657,520	1.27^−05^	0.93
1	135,592,093	4.56^−09^	0.89
1	136,856,951	5.58^−07^	0.86
1	147,891,562	1.79^−05^	0.59
1	166,623,793	2.73^−05^	0.12
1	170,453,795	6.18^−05^	0.62
2	7,930,470	6.69^−06^	0.14
2	104,416,670	1.29^−04^	0.74
3	1,436,589	5.18^−05^	0.75
3	55,172,371	1.59^−04^	0.58
3	66,036,636	5.50^−05^	0.85
5	36,974,094	3.75^−06^	0.94
5	37,008,147	6.59^−05^	0.93
5	46,265,858	2.42^−05^	0.48
6	5,440,052	2.94^−04^	0.6
6	38,543,055	4.65^−05^	0.82
6	39,345,845	1.73^−05^	0.05
6	39,682,718	1.11^−05^	0.04
6	45,103,810	6.34^−07^	0.60
7	2,045,064	1.54^−06^	0.57
7	3,007,297	7.02^−05^	0.4
7	3,054,875	1.70^−04^	0.4
7	3,852,615	2.18^−04^	0.4
7	4,035,315	1.48^−05^	0.6
7	5,372,955	4.03^−04^	0.38
7	7,123,139	1.98^−05^	0.14
7	7,139,162	1.72^−05^	0.14
7	7,146,253	4.03^−06^	0.14
7	7,165,975	1.80^−05^	0.14
7	7,176,912	1.18^−05^	0.86
10	26,917,496	7.26^−05^	0.14
10	26,940,323	1.16^−06^	0.25
11	940,827	2.59^−05^	0.8
11	5,360,569	4.84^−04^	0.36
11	6,211,604	5.43^−04^	0.37
11	7,057,578	5.16^−07^	0.76
11	7,321,582	4.58^−04^	0.71
11	8,209,704	1.22^−04^	0.33
12	6,697,076	5.59^−04^	0.92
12	12,978,667	2.05^−04^	0.82
13	115,914	3.37^−04^	0.03
13	1,601,209	1.82^−05^	0.02
13	18,085,116	3.63^−04^	0.81
17	5,328,089	2.47^−04^	0.56
19	1,435,119	5.37^−08^	0.94
22	5,854,798	3.25^−04^	0.03
22	12,682,387	8.94^−04^	0.4
23	72,964	2.20^−06^	0.95
23	2,650,159	1.16^−03^	0.14
23	5,922,028	1.04^−03^	0.71
23	5,926,679	3.73^−04^	0.29
23	5,941,776	2.76^−05^	0.28
23	5,955,710	3.44^−05^	0.28
23	5,962,837	5.42^−04^	0.72
26	293,444	1.33^−03^	0.7
26	377,952	3.79^−04^	0.72
26	5,200,221	3.61^−04^	0.01
28	352,290	1.14^−03^	0.34
28	355,120	3.80^−04^	0.74
28	1,464,794	1.29^−03^	0.64
28	2,956,468	2.07^−03^	0.6
28	2,971,481	1.95^−03^	0.69
29	2,849,936	5.98^−04^	0.05

These markers overlap with 281 genes, being 258 protein-coding, 15 long-noncoding RNAs, 3 microRNAs, 3 pseudogenes, and 2 small nucleolar RNA. The description of the genes associated with walking ability in the male line is presented in Supplementary Material 4.

Genes associated with walking ability in the male line are involved in biological processes such as cell growth (e.g., *RB1CC1* and *TNNI1*) and myostatin development (e.g., *MSTN*). Additionally, genes potentially linked to specific diseases and deformities were identified. For instance, *FN1* and *SIK3* are associated with spondyloepiphyseal dysplasia, *PADI2* is related to sclerosis, *ERBB4* is connected to amyotrophic lateral sclerosis (**ALS**), and *B3GNT2* and *BACE1* are associated with muscular dystrophy and muscular inflammation, respectively.

The enriched GO terms for walking ability are presented in Table 9. In summary, 6 biological processes and 6 cellular components are statistically associated with the genes related to walking ability in the male line.

**Table 9:** Gene ontology terms for the genes annotated for walking ability in a turkey male line population.

Term ID	Source	Gene Ontology term	*P*-value
GO:0010898	GO:BP	Positive regulation of triglyceride catabolic process	3.05^−02^
GO:0033700	GO:BP	Phospholipid efflux	3.05^−02^
GO:0034370	GO:BP	Triglyceride-rich lipoprotein particle remodeling	3.05^−02^
GO:0034372	GO:BP	Very-low-density lipoprotein particle remodeling	3.05^−02^
GO:0010896	GO:BP	Regulation of triglyceride catabolic process	4.02^−02^
GO:0050996	GO:BP	Positive regulation of lipid catabolic process	4.02^−02^
GO:0034361	GO:CC	Very-low-density lipoprotein particle	1.94^−02^
GO:0034364	GO:CC	High-density lipoprotein particle	1.94^−02^
GO:0034385	GO:CC	Triglyceride-rich plasma lipoprotein particle	1.94^−02^
GO:0034358	GO:CC	Plasma lipoprotein particle	3.83^−02^
GO:1990777	GO:CC	Lipoprotein particle	3.83^−02^
GO:0032994	GO:CC	Protein-lipid complex	4.34^−02^

Abbreviations: BP, biological process; CC, cellular component.

## DISCUSSION

Poor walking ability in meat poultry has gained increasing attention in the past few years (Kapell et al., 2012) due to the welfare and economic implications (Dawkins and Layton, 2012). The primary goal of this study was to investigate the genetic background of walking ability in female and male pure lines of turkeys. As shown in Table 2, Table 4, walking ability was moderately heritable (0.23 ± 0.01) in both populations, indicating that genetic selection can be an effective strategy for improving walking ability in commercial turkeys. Similar results were found by Abdalla et al., 2019 in a turkey population. Consequently, the inclusion of walking ability in selection programs improves welfare and production.

As a result of the intensive artificial selection, modern turkey lines weigh up to 3 times more than their wild ancestors, with relatively little change in the length of their bones and limbs (Stover et al., 2018). This is a phenomenon also observed in other poultry species selected for fast growth such as broiler chickens (Hartcher and Lum, 2020). Consequently, heavier bodies can directly impact leg health and mobility, affecting animal welfare by inflicting pain, causing changes in leg structure, posture, and quality of movement, which ultimately reduce the activity level and the capacity to display normal behaviors such as feeding and drinking (Kapell et al., 2017). This corresponds to the findings of this study as observed in Table 3, Table 5. Body weight was not only unfavorably correlated with walking ability but also with leg defects, indicating that heavier body weights were associated with leg problems and consequently poor walkability scores. This suggests that walking ability score as a composite indicator of mobility needs to be considered for breeding more robust and productive animals.

In this study, walking ability was found to be negatively genetically and phenotypically correlated with most performance traits evaluated, especially those related to the growth and development of the animals (e.g., body weight 1, body weight 2, breast meat yield, and live weight). This pattern has been previously described in other poultry studies (e.g., Kapell et al., 2017; Zhang et al., 2018; Abdalla et al., 2019). Despite this negative correlation, there is evidence that enough variability exists, allowing individuals with superior genotypes for both body weight and walking ability to coexist in a population (Quinton et al., 2011), indicating that walking ability could be suitable to add to current selection indexes without a significant impact on performance traits.

Regarding the traits related to mortality and disorders, leg defects was the only trait to show a strong unfavorable genetic and phenotypic association with walking ability. This is expected, as leg deformities can impact the locomotion and mobility of the animals, leading to worse walking ability (Kapell et al., 2017). Therefore, leg defects is a trait that affects the score classification system for walking ability (Quinton et al., 2011). Interestingly, leg defects were also shown to have a positive association with egg production. This could be explained since eggshell formation requires available calcium and this process and can affect the mineral distribution of the bone which could increase the risk of leg problems (Fleming, 2008), potentially leading to leg deformities.

For this study, we opted to analyze and present the results separately for each population due to their limited genetic connectedness and different selection goals (different traits or weights in the selection indexes). These populations represent distinct turkey groups selected for different purposes as one is a male line and the other one a female line. While some similarities exist between them, they primarily stem from specific traits considered during the selection process, albeit with differing emphasis across populations. An interesting finding is the contrasting genetic correlations between egg production and walking ability in the female and male lines. This might be because both traits were considered in the selection process for the female line but not for the male line. As a result, animals in the female line have been selected for higher egg production while maintaining good walking ability scores.

Concerning the GWAS, in both populations, multiple markers and genes were related to the walking ability traits, suggesting a polygenic nature for the trait. In the female line, the genes *CSRP2, DDX1*, and *RHBDL1* were found to be associated with walking ability through growth and cell development. This could indicate a negative pleiotropic effect occurring in the population, given the high negative genetic correlation observed among performance traits, especially body weight 1, body weight 2, and live weight. These genes have been described in the literature as promoters of muscle development (*CSRP2* – Han et al., 2019; Cui et al., 2020), cell differentiation (*DDX1* – Godbout et al., 2002), or associated with body weight (*RHBDL1* – Dadousis et al., 2021).

Two genes were found to be related to some disorder traits: *SEZ6L*, also known as Seizure Related 6 Homolog Like, which has been associated with neurological diseases in humans (Qiu et al., 2021) and behavioral locomotion and motor function in mice (Ong-Pålsson et al., 2022). Although these studies were performed in mammals, they suggest an effect of such genes on locomotion through neural activity. Additionally, the *CTSK* gene plays a role in bone resorption and potentially contributes to the regulation of bone remodeling disorders. A mutation in this gene can cause a rare autosomal recessive bone disorder called pycnodysostosis (a defect in protein cathepsin K), which leads to abnormal degradation of bone matrix, pathologic fractures, open fontanels, and sutures (Xue et al., 2011). The expression of this protein and its implications for bone structure have been studied in poultry species, including turkeys (Sosnicki and Wilson, 1991) and ducks (Zhang et al., 2019b).

Certain GO terms are directly related to turkey walking ability. For instance, GO:0001968, or fibronectin binding, refers to a glycoprotein found in extracellular connective tissue matrices associated with connecting cartilage or bone matrix to adjacent cells (Nordahl et al., 1995). Additionally, GO:0018149, or peptide cross-linking, is directly related to cartilage, contributing specifically to the mechanical stability of the cartilage (Wu et al., 1992). Femoroacetabular impingement is linked to cartilage damage (Fadul and Carrino, 2009; Speirs et al., 2013). When damage manifests in the organism, an inflammatory process may occur, directed toward a catabolic process (GO:0009057). This process could lead to cases of hip impingement and the development of osteoarthritis (Chinzei et al., 2016). In such a scenario, if the animals are affected by these issues, they would likely exhibit a poor walking ability score and other problems associated with mobility and locomotion.

For the male line, a higher number of markers were shown to be associated with walking ability. Among these, some of the genes were found to be related to the process of cell growth. For example, the *RB1CC1* gene has been identified as a marker associated with obesity in chickens (Cheng et al., 2019), and maturation of embryonic musculoskeletal cells in humans (Chano et al., 2002). Similarly, the *TNNI1* gene has been identified as playing an important role in duck muscle fiber development and meat quality, with high expression levels in leg muscle during early development (Shu et al., 2019). Additionally, *MSTN*, which has been associated with growth traits in chickens, can regulate skeletal muscle growth (Zhang et al., 2019a). This parallels the situation observed in the female line, where genes selected for growth could have a negative pleiotropic effect on the walking ability of the animals.

Some of the candidate genes found to be related to walking ability in turkeys have also been previously described in the literature to be associated with pathologies such as spondyloepiphyseal dysplasia, sclerosis, and dystrophia. The *FN1* gene, or Fibronectin1, is described as multifunctional glycoproteins that play an essential role in chondrogenic and osteogenic differentiation (Aladal et al., 2022). The functions of the *FN1* gene are linked to the extracellular matrix and directly correlated with bone function (de Oliveira Peixoto et al., 2019). The *SIK3* gene, or SIK family kinase 3, enables ATP binding activity, magnesium ion binding activity, and protein serine/threonine kinase activity. The *FN1* gene has also been studied as one of the major genes responsible for osteoarthritis in some species, such as mice (Yahara et al., 2016), and even in humans (Csukasi et al., 2018). Some studies show that such genes could be related to a rare recessive disease known as spondyloepiphyseal dysplasia in humans (Baratang, 2018; Yip et al., 2019). This disease affects matrix metalloproteinase 13, which plays a crucial role in bone formation and has been described in the literature as important component for turkey bone formation (Simsa et al., 2007).

The *PADI2* gene, or peptidyl arginine deiminase gene, is responsible for catalyzing an enzyme that converts arginine residues to citrulline residues in the presence of calcium ion (Ying et al., 2009). Studies have indicated that the aberrant activation of *PADI2*, a gene expressed throughout the nervous system, is likely associated with the development of neuropsychiatric diseases characterized by neurodegenerative processes, such as Alzheimer's disease and multiple sclerosis (Watanabe et al., 2009). On the other hand, the *ERBB4* gene, or erb-b2 receptor tyrosine kinase 4, is involved in the pathogenesis of amyotrophic lateral sclerosis by a mutation that reduces autophosphorylation on ErbB4 upon neuregulin stimulation (Takahashi et al., 2013).

The *B3GNT2* gene, or beta-1,3-N-acetylglucosaminyltransferase family, is the priming enzyme for the LARGE-dependent functional glycosylation of dystroglycan, which is crucial for extracellular matrix protein binding (Praissman et al., 2014). Defects in dystroglycan can account for most cellular pathologies and disorders, as muscular dystrophy causes progressive weakness and loss of muscle mass (Martin, 2005). Muscular dystrophy has already been described as a problem in domestic turkeys as a hereditary histopathology (Schmitz and Harper, 1975). The *BACE1* gene, or Beta-Secretase 1, encodes a member of the peptidase A1 family of aspartic proteases. *BACE1* inhibits inflammation and fatty acid oxidation caused by palmitate in myotubes (Botteri et al., 2018) leading to inflammation of muscles and myotube atrophy (de Hart et al., 2023).

Related to the GO in the turkey's male line, most of the term elements seem to be related to lipid metabolism. This might be explained by the fact that turkeys selected for fast growth could allocate more nutrients for skeletal muscle and fat deposition instead of bone development (Soyalp et al., 2023). This influence on skeletal homeostasis is exerted through fatty acid oxidation in osteoblasts, the cells responsible for creating new bone and facilitating the growth and development of existing bones (Alekos et al., 2020). Some studies have already reported the effect of lipid metabolism on bone growth and development in broiler chicks (Watkins et al., 1997; Fan et al., 2021). This underscores the importance of lipid metabolism in bone health, as they may result in changes in bone homeostasis and an increase in osteopathy, such as osteoarthritis and fractures (Wang et al., 2022).

As observed in this study, walking ability emerges as a trait with potential for selective improvement, deserving consideration in both animal welfare and production. Being a polygenic trait, it involves a complex interplay of mechanisms, encompassing multiple metabolic processes and genes influencing the trait expression. Notably, intriguing findings in this study prompt the need for further investigations, particularly given the negative correlation observed between walking ability and most production traits. The identification of similar genes associated with both walking ability and these traits suggests the occurrence of pleiotropic effects within the populations.

Additionally, to enhance the understanding of these traits and their influence on walking ability, future studies should focus on evaluating the health and structure of bones and cartilage. The intricate involvement of numerous genes and mechanisms associated with walking ability suggests potential impacts on these anatomical features, warranting comprehensive investigations for a more thorough comprehension of the trait dynamics.

## DISCLOSURES

The authors declare no conflicts of interest.

## Appendix Supplementary materials

Image, application 1

Image, application 2

Image, application 3

Image, application 4

## Footnotes

Supplementary material associated with this article can be found in the online version at doi:10.1016/j.psj.2024.103779.
